# Multivariate analysis of craniodental morphology in mouse-eared bats (Chiroptera, Vespertilionidae, *Myotis*) from Vietnam

**DOI:** 10.3897/BDJ.12.e122597

**Published:** 2024-06-27

**Authors:** Huong Yen Vu, Tuan Hai Bui, Trung Thanh Hoang, Kim Luong Vu, Truong Son Nguyen

**Affiliations:** 1 Department of Zoology & Conservation, Faculty of Biology, University of Science, Vietnam National University, Hanoi, Vietnam Department of Zoology & Conservation, Faculty of Biology, University of Science, Vietnam National University Hanoi Vietnam; 2 Institute of Genome Research, Vietnam Academy of Science and Technology (VAST), Hanoi, Vietnam Institute of Genome Research, Vietnam Academy of Science and Technology (VAST) Hanoi Vietnam; 3 Vietnam National Museum of Nature, VAST, Hanoi, Vietnam Vietnam National Museum of Nature, VAST Hanoi Vietnam; 4 Department of Vertebrate Zoology, Institute of Ecology and Biological Resources, VAST, Hanoi, Vietnam Department of Vertebrate Zoology, Institute of Ecology and Biological Resources, VAST Hanoi Vietnam

**Keywords:** skull variation, PCA, comparison, dentition, small mammal

## Abstract

This study conducted biostatistical multivariate analyses on 23 craniodental morphological measurements from 209 specimens to study interspecific variations amongst 15 bat species of the genus *Myotis* in Vietnam. Univariate and multivariate analyses demonstrated that the studied species can be divided into four groups as follows: extra-large-sized species (*M.chinensis*), large-sized species (*M.pilosus*, *M.indochinensis* and *M.annectans*), medium-sized species (*M.altarium*, *M.hasseltii*, *M.montivagus*, *M.horsfieldii*, *M.ater*, *M.laniger* and *M.muricola*) and small-sized species (*M.annamiticus*, M.aff.siligorensis, *M.rosseti* and *M.alticraniatus*). Our data revealed that the main craniodental features contributing to the variations in distinguishing *Myotis* species are the width of the anterior palatal, least height of the coronoid process, length of the upper and lower canine-premolar, zygomatic width and width across the upper canines and lower premolar-molar length. Based on patterns of morphological differences, we conducted comparisons between morphometrically closely resembling species pairs and further discussed additional characteristics that are expected to support the taxonomy and systematics of Vietnamese *Myotis* bats.

## Introduction

The vespertilionid bats of the genus *Myotis*, with approximately 139 extant species, are widely distributed throughout the world, including Vietnam (Simmons and Cirranello 2024). *Myotis* is considered a taxon that has not developed particular characteristics, retaining primitive dentition (Gunnell et al. 2012). Like most vespertilionids, *Myotis* bats possess exaggerated morphological specialisations, such as a greatly enlarged cochlea, associated with advanced echolocating abilities (Simmons et al. 2008). Bats of the genus *Myotis* range in size from relatively small to large amongst “typical” vespertilionidae, with a relatively narrow ear and the length of which always exceeds its width. The external morphologies exhibit distinctive features, including a straight, narrow and typically pointed profile of the tragus and ear pinnae that are not funnel-shaped, but instead, lightly folded along the posterior margin (Kruskop 2013b). The muzzle is either covered in fur or occasionally nearly devoid of hair. The wings range from broad to moderately narrow, with metacarpals nearly equal in length, with the fifth metacarpal slightly shorter than the third and fourth. The hind foot size and the pattern of attachment of the wing membrane to the leg display the most significant variability (Findley 1972 and Kruskop 2013b). Mouse-eared bats have the following particular dentition formula: I^2/^_3_, C^1/^_1_, P^2-3/^_2-3_, M^3/^_3_ × 2 = 34–38 (Tate 1941 and Kruskop 2013b). The first upper and lower premolars (P^2^, p_2_) maintain a simple structure with no significant reduction and are consistently present within the tooth rows. P^3^ and p_3_ exhibit a similar shape but vary in size; in the maxilla, they are notably smaller than P^2^ and p_2_. In certain *Myotis* species, P^3^ and p_3_ may protrude from the axis of the tooth rows or be absent. Upper molars feature a well-developed mesostyle and a reduced hypocone, which is consistently present; in some cases, they may also possess paraconules. Lower molars are myotodont type in most species; the upper outer incisor is accompanied by larger supplementary cusps than the inner one, while the canine lacks any supplementary cusps (Kruskop 2013b).

In Vietnam, 72 species of vespertilionoid bats have been discovered, of which 19 species belong to the genus *Myotis*, including: *Myotisaltarium*, *M.alticraniatus*, *M.annamiticus*, *M.annatessae*, *M.ancricola*, *M.annectans*, *M.ater*, *M.chinensis*, *M.formosus*, *M.hasseltii*, *M.horsfieldii*, *M.indochinensis*, *M.laniger*, *M.montivagus*, *M.muricola*, *M.phanluongi*, *M.pilosus*, *M.rosseti* and *M.rufoniger* (Kruskop 2013b andMoratelli et al. 2019). Since the first record of the *Myotis* in Vietnam (Morice 1875), the systematic complexity and inconsistency within the genus *Myotis* have been documented by various and sometimes contradictory reports (Pousargues 1904, Menegaux and Auguste 1906, Osgood 1932, Kuznetsov and Rozhnov 1998, Bates et al. 1999, Benda and Tsytsulina 2000, Kruskop and Tsytsulina 2001, Borisenko et al. 2009, Nguyen et al. 2013, Kruskop 2013a, Kruskop and Borisenko 2013, Csorba et al. 2014, Kruskop et al. 2018, Vu et al. 2018, and Ruedi et al. 2021).

Many *Myotis* species exhibit complex morphological and genetic characteristics that warrant further research. Prior to 2008, species classification within the genus *Myotis* primarily relied on external morphological traits such as fur colour, forearm length, tibia length, hind-foot length, ear length, the feature attachment of the wing membrane to the leg, characteristics of the calcar lobe in the wing membrane and craniodental morphology. Due to the similarity of some morphological characteristics and the complexity of molecular analysis amongst closely-related *Myotis* bats (Kruskop et al. 2018 and Ruedi et al. 2021), species identification becomes problematic. Therefore, assessing morphological variation within *Myotis* is indispensable prior to conducting genetic analysis to accurately determine their taxonomic positions. In this study, we first conducted univariate and multivariate analyses to determine morphometric variations in craniodental morphology and discussed interspecific variation patterns in relation to species identification.

## Material and methods

### Measurements

**Figure 1.**
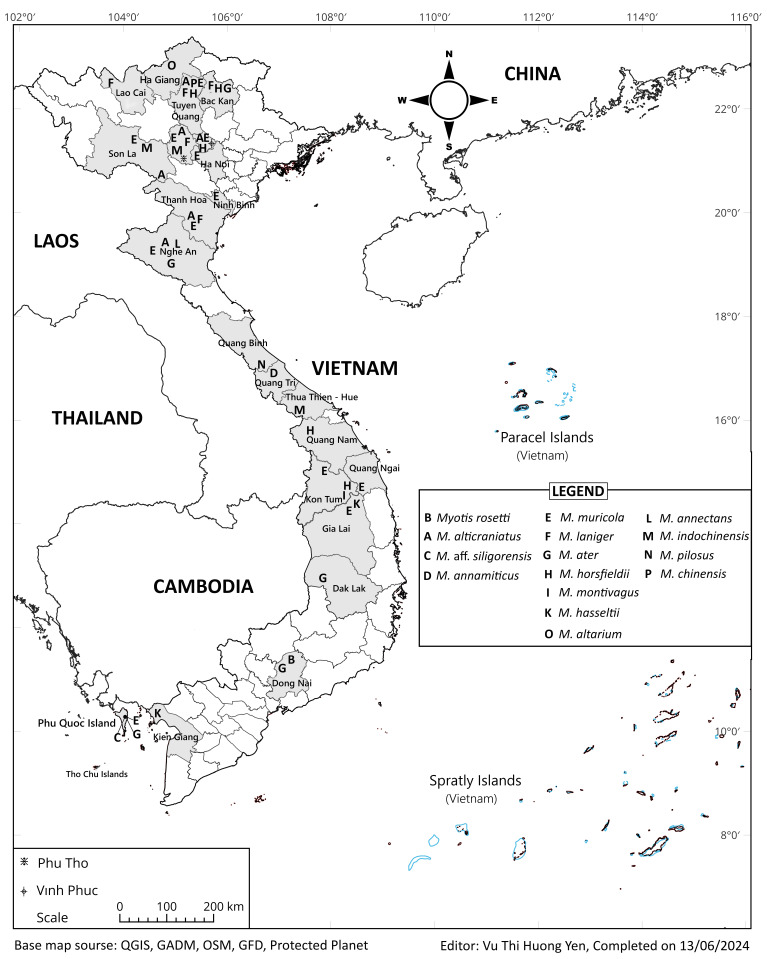
Map showing the localities of *Myotis* spp. examined in this study from 25 localities in 21 geographical provinces of Vietnam. Base map source: QGIS, GADM, OSM, Protected Planet.

**Figure 2.**
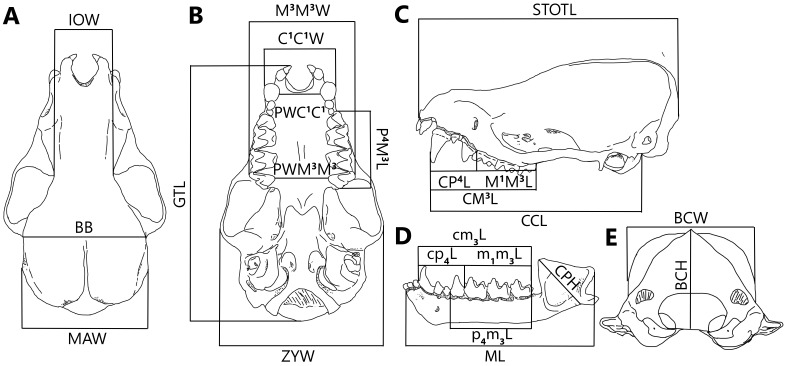
Dorsal (A), ventral (B), lateral (C), posterior (D) views of the cranium; Lateral (E) views of mandible displaying craniodental measurements.

The present study was implemented using a total of 209 skull specimens from the mouse-eared bats of genus *Myotis*, which were collected from 25 localities in 21 provinces of Vietnam (Fig. 1, Suppl. material 1). All adult specimens have been deposited in the Vertebrate Zoology Department, Institute of Ecology and Biological Resources (IEBR-VAST). Most specimens were identified using the combination of external morphology and craniodental morphology following *Kruskop (2013b), Nguyen et al. (2013)*, *Vu et al. (2018)* and *Moratelli et al. (2019)*, while the classification of 29 *Myotis*’ specimens was confirmed by COI gene sequence analysis. Our study analyses were conducted on craniodental measurements, which were effectuated by measuring under a dissecting microscope (SMZ 745, Nikon, Japan) with electronic digital calliper (NTD12-15PMX, Mitutoyo, Japan) to the nearest accuracy of 0.01 mm. The 23 craniodental characteristics were examined following Nguyen et al. 2015a and Nguyen et al. 2016 (Fig. 2, Table 1).

### Statistical analyses

Minimum, maximum, mean values, standard deviations and interquartile range (IQR) for 23 measurements were calculated using Microsoft^®^ Excel version Office 2021 (Microsoft, Redmond, WA, USA). Multivariate analysis of variance (MANOVA) using log-transformed craniodental measurements indicated non-significant sexual-dimorphism differences for five out of 15 *Myotis* species with sufficiently large sample sizes. Thus, our study was performed on all specimens without sexual discrimination in statistical analyses. Univariate analyses and multivariate analyses of craniodental morphology using Principal Component Analysis (PCA) were conducted to evaluate correlations between interspecific morphometric variations of Vietnamese *Myotis* bats. Differences in the mean values were examined by analysis of variance (One-way ANOVA) and Tukey’s pairwise test of variance (significant at p < 0.05). Pairwise comparisons were carried out using F and t-tests (P < 0.05) amongst taxa for difference comparison. All these analyses were performed using the PAST software ver.4.13 (Hammer et al. 2001). All the measurements are in mm.

## Results

### Differentiation of interspecific craniodental morphological appearance amongst the groups

**Figure 3.**
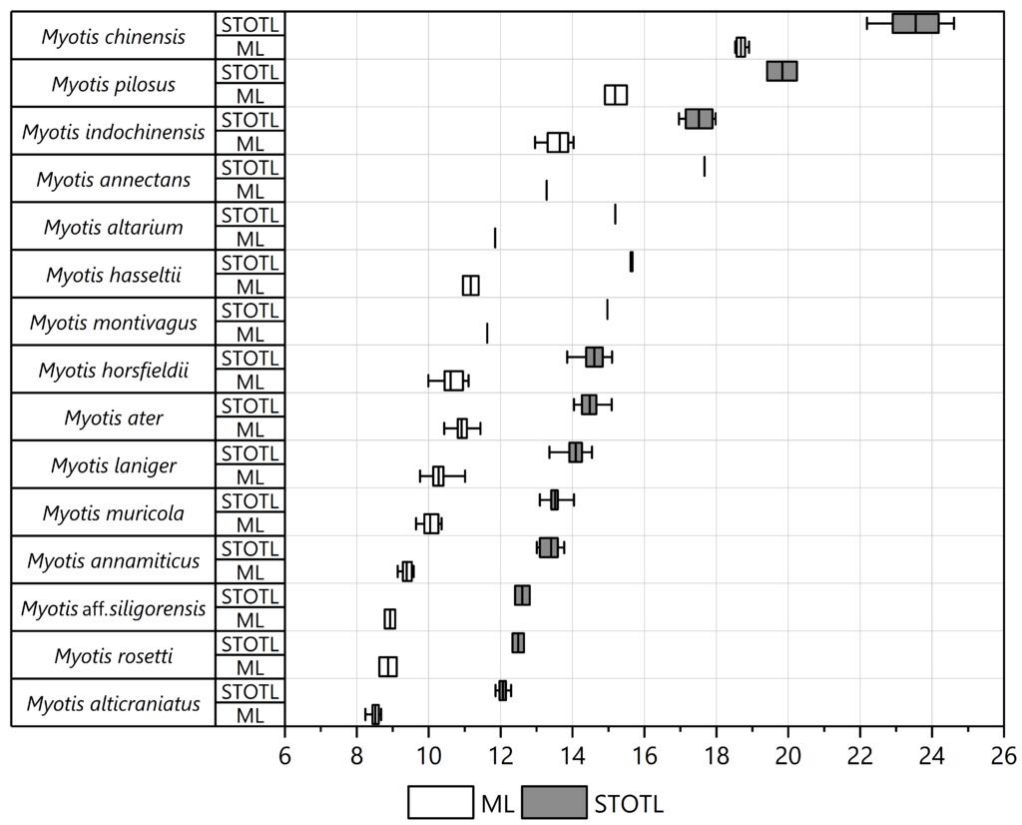
Boxplots showing range (minimum value to maximum value in horizontal line), mean value (in vertical bar), IQR (in rectangle box) of STOTL and ML measurements of 15 *Myotis* species.

**Figure 4.**
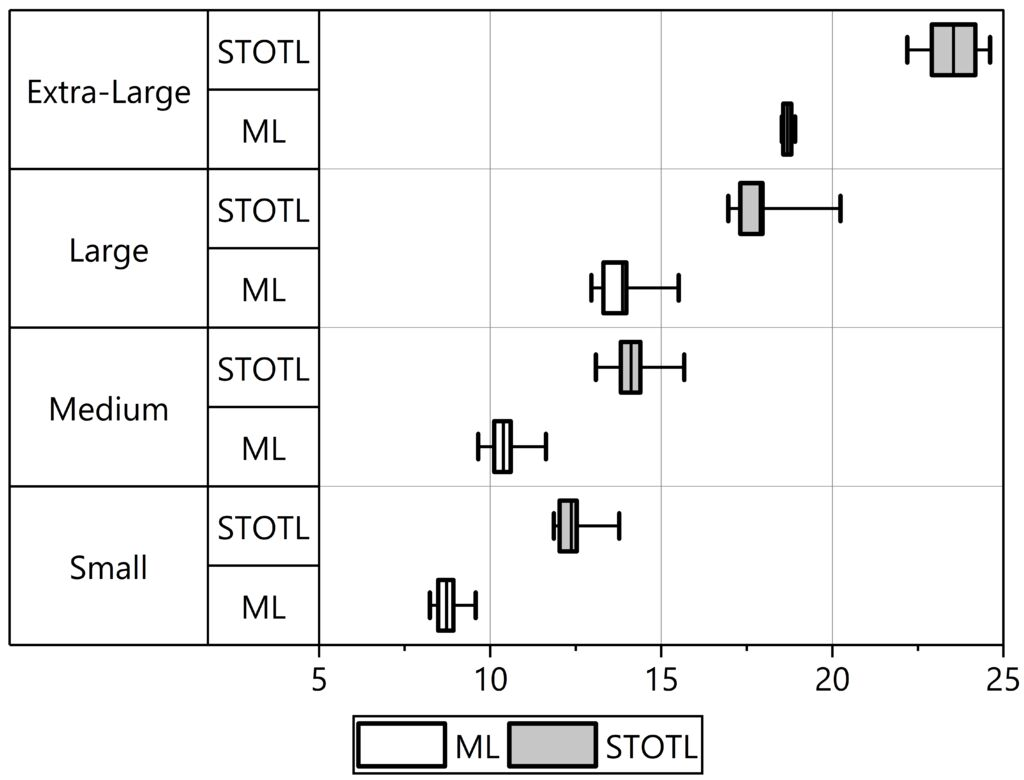
Boxplots showing range (minimum value to maximum value in horizontal line), mean value (in vertical bar), IQR (in rectangle box) of STOTL and ML measurements of four Vietnamese *Myotis* groups.

Descriptive statistics for craniodental measurements are presented in Table 2. The differences amongst taxa in all craniodental characteristics were detected by one-way ANOVA (p < 0.05). The largest standard deviations were found in STOTL and GTL related to cranial size by length and ML related to mandible size by length, indicating significant variability within these parameters. Based on comparing STOTL and ML measurements (Fig. 3 and Fig. 4), 15 *Myotis* species of Vietnamese mouse-eared bats recorded in this study could be divided into four groups with distinct sizes precisely:

- Group S (small size, STOTL: Mean = 12.45, range = 11.86–13.77, ML < 9.58 mm) includes four species of *M.alticraniatus*, *M.rosseti*, M.aff.siligorensis and *M.annamiticus*.

- Group M (medium size, STOTL: 14.123, 13.09–15.67, ML: 10.394, 9.65–11.85) includes seven species in the descending order: *M.altarium*, *M.hasseltii*, *M.montivagus*, *M.horsfieldii*, *M.ater*, *M.laniger* and *M.muricola*.

- Group L (large size, STOTL: 17.89, 16.96–20.24, ML: 13.87, 12.96–15.51) comprises three distinctive species: *M.pilosus*, *M.indochinensis* and *M.annectans*.

- Group XL (extra-large size, STOTL: 23.54, 22.19–24.61, ML: 18.68, 18.52–18.91) comprises one species, *Myotischinensis*, which was considered the greatest and most noteworthy difference in size compared to other *Myotis* species of the aforementioned groups, as indicated by measurements.

**Figure 5.**
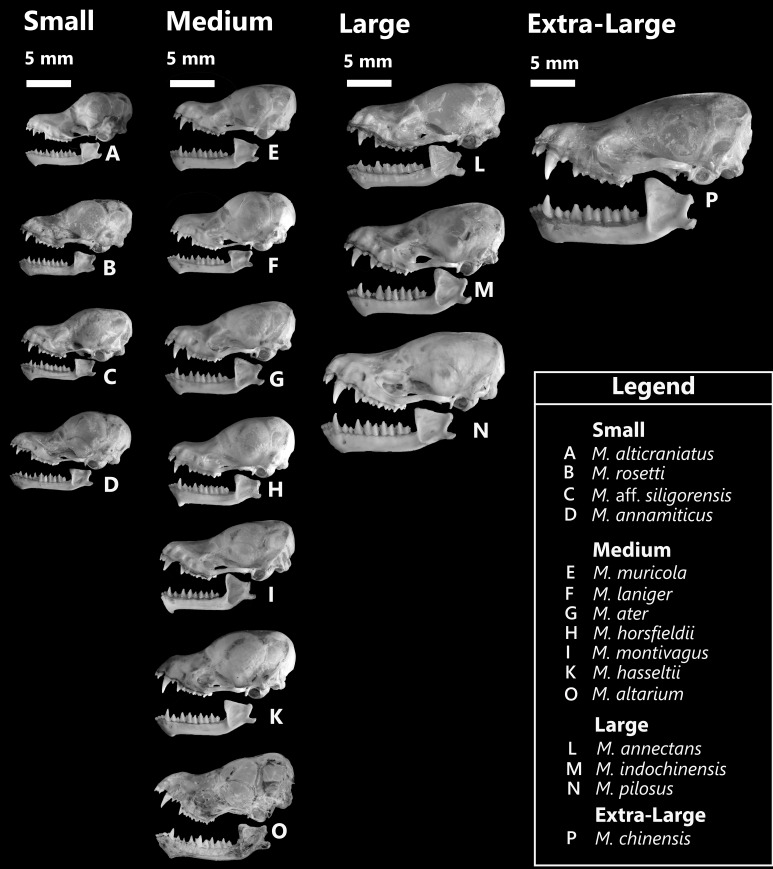
Cranium and mandible morphology of four Vietnamese *Myotis* groups.

The differences amongst these four groups can be easily detected by direct observation of the appearances of the skulls (Fig. 5) and are determined to be significant, based on T-test and W-test.

The factor loadings for log-transformed measurements are presented in Table 3. The first principal component (PC 1) explaining 91.4% the total variance for all specimens was interpreted to represent size and shape variation, because all character factor loadings were positive and showed higher values in P^4^M^3^L, ZYW, C^1^C^1^W, CPH, cm_3_L, cp_4_L, p_4_m_3_L and m_1_m_3_L (Table 3). The second principal component (PC 2) assessed 3.7% of the variances for all specimens with higher absolute values in CPH (negative, n), PWC^1^C^1^ (positive, p), CP^4^L (p), cp_4_L (p) and ZYW (n) (Table 3). The highest factor loadings for both PCs were CPH, ZYW and cp_4_L.

**Figure 6.**
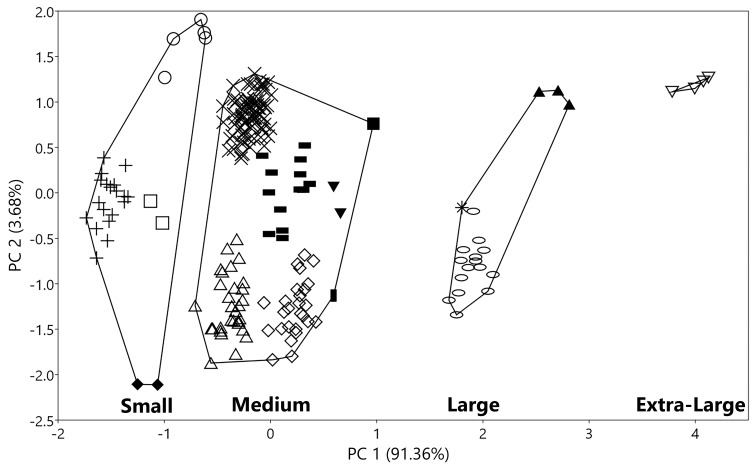
Bivariate scatterplots of the first and second principal component scores, based on log-transformed craniodental measurements in four Vietnamese *Myotis* groups.

The PC scatterplots distinguished large- and extra-large-sized *Myotis* being greater compared to two smaller *Myotis* groups (Fig. 6). The MANOVA further indicated differences between the smaller-sized bats (M and S *Myotis* groups) compared to the two larger ones (L and XL *Myotis* groups) (Wilk’s lambda = 4.45E-250, p = 2.02E-59, p < 0.001). Particularly, PC 1 has a high correlation with STOTL. Tukey's pairwise test distinguished significant differences between the larger taxa and the smaller taxa (significant comparison = 8.76E-06, P < 0.05).

**Figure 7.**
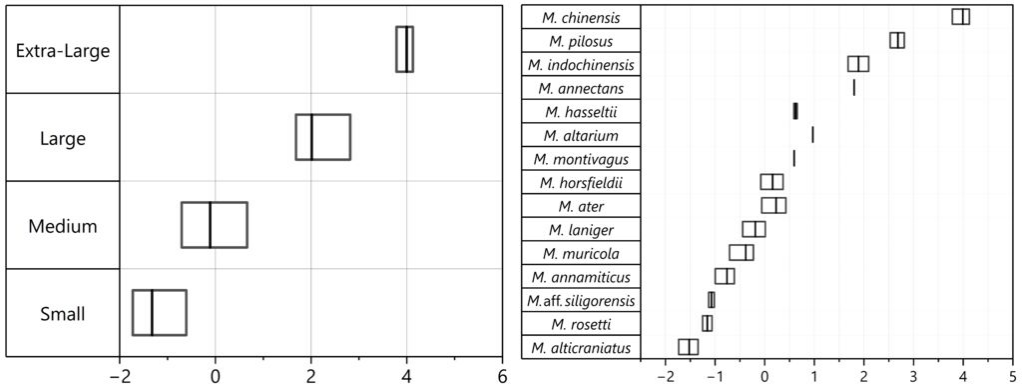
Range (minimum value to maximum value in horizontal rectangle box) and mean value (in vertical bar) of PC 1 scores for log-transformed measurements of four Vietnamese *Myotis* groups (left) and 15 Vietnamese *Myotis* species (right).

Bivariate scatterplots of PC 1 and PC 2 completely separated the four groups by considering only the PC 1 values (Fig. 6) (t-test, ANOVA, Turkey’s pairwise comparison: p < 0.05). Statistical analysis revealed noteworthy differences amongst the four mouse-eared bat groups (F-test, p < 0.001). The t-test exhibited significant variation between Group L and Group XL in PC 1 scores (t-test, t = 11.981, p < 0.05). Group L was further distinguished from Group M (t-test, t = 33.136, p < 0.05). Although the PC 1 values slightly overlap between *M.muricola* of group M and *M.annamiticus* of group S (Fig. 6, Fig. 7, Table 4), the cranium’s morphology showed that these two species were distinct from each other (Fig. 5). Furthermore, One-way ANOVA indicated Group M substantially differed from Group S (F = 487.4, *p* < 0.001, Tukey’s test, *P* < 0.05, t-test, t = 22.076, *p* < 0.05). PC 2 did not show significant differences amongst the four groups (MANOVA: F = 3.456, *p* = 0.017, *p* > 0.001).

The range and mean value of PC 1 scores showed craniodental-sized variation of each mouse-eared bat in Fig. 7 (right) and in which the PC 1 was greatest in the distinctive species *Myotischinensis*, followed by the large-sized *Myotis*: *M.pilosus*, *M.indochinensis* and *M.annectans*. The PC 1 scores of *M.pilosus* were significantly greater than those of both *M.indochinensis* and *M.annectans* (One-way ANOVA, *p* = 7.54E-09), whereas these values of *M.indochinensis* and *M.annectans* overlapped (one-way ANOVA, *p* = 0.739). The values for both of these species significantly surpassed those for the other smaller one (t-test, t = 21.405, *p* = 2.253E-54). The PC 1 scores for *Myotis* of Group M were greatest in *M.altarium*, distinctively separating the other medium-sized *Myotis* (Fig. 7 right). The PC 1 scores of *M.hasseltii* completely overlapped with those of *M.montivagus* (Mann-Whitney’s pairwise, differences *P* = 0.54); its overall overlapping tendency similarly arose in the two smaller species: *M.horsfieldii* and *M.ater* (t-test, t = 1.561, *p* = 0.127), while *M.laniger* had non-significant differences and only PC 1 score partially intersected with *M.muricola* (Fig. 7) (Mann-Whitney *U* = 263, *p* = 1.175E-10). Amongst the four small-sized *Myotis*, the lowest PC 1 coefficient was observed in *M.alticraniatus*, which had a distinctive value against the three larger-sized species: *M.annamiticus*, *M.rosseti* and M.aff.siligorensis (t-test, t = 9.472, *p* = 6.48E-10), with *M.annamiticus* being pointedly greater than *M.rosseti* and M.aff.siligorensis (One-way ANOVA, *P* < 0.05; t-test, t = 3.485, *p* = 0.01). These two *Myotis* considerably overlapped in PC 1, but the values of M.aff.siligorensis (mean = –1.074, range = [–1.13, –1.018]) were indicated to be slightly larger than those of *M.rosseti* (mean = –1.157, range = [–1.252, –1.063]) (Fig. 7 right, Table 4).

**Figure 8.**
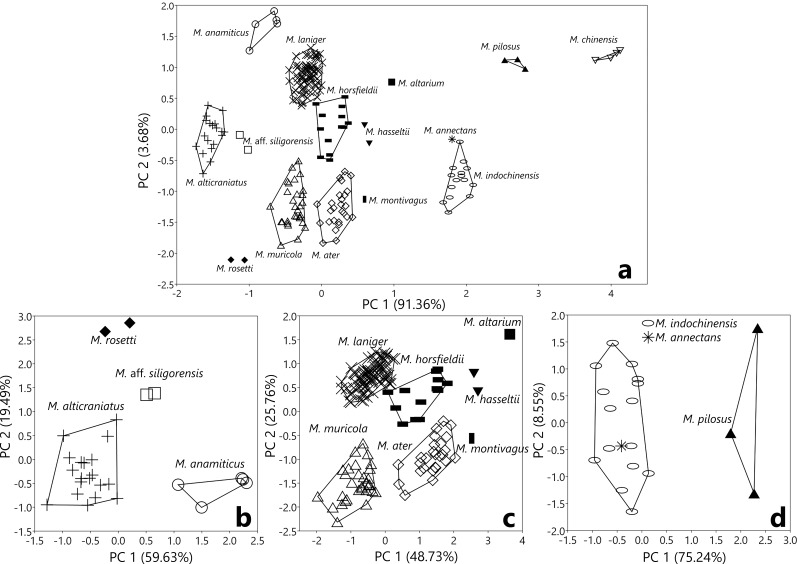
Bivariate scatterplots of the first and second principal component scores, based on log-transformed craniodental measurements for: (**a**) 15 mouse-eared bats; (**b**) small-sized *Myotis*; (**c**) medium-sized *Myotis*; (**d**) large-sized *Myotis*.

The complete separation amongst the studied species is further clarified in the plots in Fig. 8a. The sizes of the 15 *Myotis* species, considering the interference of two PC scores, were in the following order: *M.chinensis*, *M.pilosus*, *M.indochinensis*, *M.annectans*, *M.altarium*, *M.hasseltii*, *M.montivagus*, *M.horsfieldii*, *M.ater*, *M.laniger*, *M.muricola*, *M.annamiticus*, M.aff.siligorensis, *M.rosseti* and *M.alticraniatus*. Craniodental morphometric variations amongst the 15 studied bat species were observed from the higher factor loadings of PC 1 and PC 2 to be distinct in the zygomatic arch, canines, first upper and lower premolars, molars and the coronoid process characteristic. In contrast to PC 1, the PC 2 score showed non-conspicuous differences; the interspecific variations in the braincase were not clearly different when considering this score. Only that of *M.annamiticus* might be distinct, while the other *Myotis* remarkably overlapped (PC 2 score axis, Fig. 8a) (one-way ANOVA, F = 15.22, *p <* 0.0001). However, to eliminate the influence of grouping by STOTL and ML, interspecific variations amongst taxa were analysed by groups (S, M and L) (Fig. 8b, c, d).

### Interspecific variation comparisons within group

#### Group S

The PCA result of group S (Fig. 8b) showed the significant differences in the craniodental measurements distinguishing these four small-sized *Myotis* species into separated and non-intersecting clusters. PC 1 accounted for 59.6% of the variances, with all character factor loadings being positive and the highest score recorded in cp_4_L followed by smaller values which were detected in CP^4^L, PWC^1^C^1^ and CPH (Table 3). PC 2 explained 19.5% of the variances, with higher factor loadings for CPH, IOW, ZYM (positive) and CP^4^L, cp_4_L (negative) (Table 3).

One-way ANOVA detected significant differences amongst all four small *Myotis* species (*p* < 0.001) in PC 1, which represented the apparent distinctions between *M.alticraniatus* and M.aff.siligorensis (One-way ANOVA, F = 23.19, *p <* 0.001), *M.alticraniatus* and *M.annamiticus* (F = 163.9, *p <* 0.001), between *M.annamiticus* and 2 species of *M.rosseti*, M.aff.siligorensis (F = 26.24, *p <* 0.001) (Fig. 8b). Although the difference was not observed between *M.alticraniatus* and *M.annamiticus* considering the PC 2 axis (t test, t = 1.2, *p* = 0.243), significant difference was found pairwise between these two species versus *M.rosseti* and M.aff.siligorensis (One-way ANOVA, *p <* 0.001 for each pair).

#### Group M

The scatterplot of PCA for medium-sized *Myotis* between PC 1 and PC 2 displayed the absolute differences and plainly separating these seven sub-groups within non-intersecting clusters (Fig. 8c). PC 1 represent mostly craniodental variations, elucidating 48.7% of the interspecific variances. All character factor loadings were positive, with the highest value recorded in CPH, though smaller scores were recorded in C^1^C^1^W and ZYW (Table 3). PC 2 defined 25.8% of the variances, with high factor loadings for BCW and PWC^1^C^1^ (p) likewise indicating the highest loading value and CPH (n) (Table 3).

One-way ANOVA test and Mann-Whitney pairwise test indicated significant differences between pairwise species according to PC 1 and PC 2, as shown in the following Table 5:

One-way ANOVA with PC 1 signified the distinct differences between *M.muricola* and *M.ater* (F = 407.3, *p <* 0.01), *M.muricola* and *M.horsfieldii* (F = 150, *p <* 0.01), *M.laniger* and *M.ater* (F = 512.3, *p <* 0.01), *M.laniger* and *M.horsfieldii* (F = 172.3, *p <* 0.01) (Fig. 8c). The *M.altarium*, *M.hasseltii* and *M.montivagus* were distinguished from the others of medium-sized *Myotis* by their actual larger craniodental scopes (Mann-Whitney pairwise with *p*-value = 0.000665), although no difference was observed between the two species *M.hasseltii* and *M.montivagus* (One-way ANOVA, F = 1.012, *p* = 0.42). The analogous tendency was verified correspondingly when considering the relationship between *M.horsfieldii* and *M.ater*, which completely overlapped (t-test, t = 1.89, *p* = 0.07; Mann-Whitney pairwise with raw *p*-value = 0.175, *U* = 123; One-way ANOVA, F = 3.59, *p* = 0.12). Further, two smaller bats, *M.muricola* and *M.laniger*, slightly overlapped (Fig. 8c), but most *M.laniger* specimens are larger in PC 1 than the other *Myotis*. Otherwise, PC 2 demonstrated appreciable differences between each pair of species as *M.laniger* and *M.ater*, *M.horsfieldii* and *M.muricola*, *M.hasseltii* and *M.montivagus*, between *M.hasseltii* and 2 species of *M.muricola*, *M.ater* (One-way ANOVA, *p <* 0.01 for each pair). Nonetheless, there was nearly an overlap between *M.horsfieldii* and *M.ater* (F = 1.039, *p* = 0.892); however, no differences were observed amongst the populations of *M.laniger*, *M.horsfieldii* and *M.hasseltii* (PC 2 axis, Fig. 8c) (One-way ANOVA, F = 0.378, *p* = 0.686) which were analogous to the three species (*M.muricola*, *M.ater* and *M.montivagus*).

#### Group L

Scatter plots between PC 1 and PC 2, based on PCA results, showed a clear separation of the *M.pilosus* sub-group with the *M.indochinensis* sub-group and a point of *M.annectans* completely mosaic within it (Fig. 8d).

In PCA, PC 1 explained 75.24% of the interspecific variation in craniodental measurements, but this consequence arose because of the completely different cranium and mandible sizes of *pilosus* from the other two species (Fig. 3, Table 2). Character factor loadings for PC 1 were positive, with the highest values in PWC^1^C^1^, followed by loading factors in cp_4_L and cm_3_L (Table 3). PC 2 explained 8.55% of the differences, with high loadings for CPH, cp_4_L, CP^4^L (positive) and PWC^1^C^1^ (negative), while the CP^4^L measurement showed the highest factor loading value (Table 3). Considering the correlation between two PC values, PC 1 indicated the distinctive differences between *M.pilosus* against *M.indochinensis* and *M.annectans* (One-way ANOVA, F = 145.7, *p <* 0.01), but no differences were observed between the populations of *M.indochinensis* and *M.annectans* in both PC 1 and PC 2. PC 2 exhibited non-significant differences amongst these three species (One-way ANOVA, F = 0.257, *p* = 0.776) (Fig. 8d, Table 4).

## Discussion

Dentition characteristics, coronoid process and braincase height have often been mentioned as informative diagnostic features in *Myotis* species and in other bats and small mammals such as rodents and insectivores. The associated craniodental measurements have been indicated to be suitable for species discrimination, alongside external morphological characters (Borissenko and Kruskop 2003, Vuong et al. 2015, Nguyen et al. 2015b, Nguyen et al. 2016, Vuong et al. 2017, Vu et al. 2018, Bui et al. 2020, Zachos 2020 and Esquivel et al. 2021). Taxonomy of cryptic taxa, based on morphology and craniodental morphometrics, are consequently essential, even in the age of genetics. In our study, multivariate analyses clarified 15 interspecific distinctions and discrimination of four groups. However, species of each group also exhibit both similar characteristics, which are frequently misclassified and distinct patterns, which facilitate easy classification. This multivariate analysis study is implemented to clarify interspecific craniodental variations for each *Myotis* group throughout Vietnam, revealing varied craniodental morphometric characteristics for each group’s interspecific traits. These findings can assist in classifying this complex group of *Myotis* species prior to conducting molecular analyses to accurately determine their taxonomic positions.

Ospina-Garcés et al. (2016) and Ospina-Garcés and Arroyo-Cabrales (2018) have demonstrated that cranial morphological characteristics, particularly the size and shape of the coronoid process, are directly associated with the bite force and diet of *Myotis* species. The differences in the size and skull shape of vesper bats related to the prey size, prey hardness, amount and frequency of eating have been discussed by many authors, such as Freeman 1981, Schmid et al. (1993), Fenton and Bogdanowicz (2002), Gannon and Rácz (2006), Postawa et al. (2012), Ghazali and Dzeverin (2013), Görföl et al. (2014), Nguyen et al. (2015a), Fenton and Simmons (2016), Nguyen et al. (2016), Ospina-Garcés et al. (2016), Ospina-Garcés and Arroyo-Cabrales (2018), Moratelli et al. (2019) and Ospina-Garcés et al. (2021). Craniodental morphometric variations amongst four groups as well as 15 species of Vietnamese mouse-eared bat species in our study were observed from the higher factor loadings of PC 1 and PC 2: CPH, ZYW, cp_4_L, CP^4^L, p_4_m_3_L, cm_3_L, PWC^1^C^1^ and C^1^C^1^W, indicating distinct features in the zygomatic arch, canines, first upper and lower premolars, molars, besides the characteristics of the coronoid process.

### Small-sized Myotis

In group S, despite STOTL and ML representing *M.alticraniatus* as the smallest species, our PCA detected that CP^4^L and cp_4_L contributed the most differences, indicating that *M.rosseti* had the smallest canine-premolar lengths. This can be explained by *M.rosseti* being the only species of genus *Myotis* that lacks the third premolars on both the maxilla and mandible. Likewise, CP^4^L displayed that M.aff.siligorensis was partially smaller than *M.alticraniatus* due to the lack of third upper premolars, leading to a significant difference from *M.rosseti*. In contrast, CPH was distinctly larger in *M.rosseti*, although it was observed to be smaller in the remaining three *Myotis*. *Myotisrosseti* was characterised by having a larger CPH/cp_4_L and IOW/cp_4_L ratio compared to the other three small-sized species (Table 2). Additionally, CPH and PWC^1^C^1^ in M.aff.siligorensis specimens were greater than in *M.alticraniatus* (t-test, *p* < 0.001), while M.aff.siligorensis differed from others by having a smaller CP^4^L measurement and a nearly overlapping cp_4_L measurement.

In this study, two specimens of Myotisaff.siligorensis collected on Phu Quoc Island (Kien Giang Province) were examined. Due to abnormalities in the dentition structure and craniodental characteristics, these specimens were listed in "*siligorensis* species complex" (Tiunov et al. 2011, Kruskop 2013b, Moratelli et al. 2019, Ruedi et al. 2021) and provisionally classified as Myotisaff.siligorensis. However, according to Ruedi et al. (2021), *M.siligorensis* must be considered as a distinctive species on its own, with its distribution range likely restricted to Central and Eastern Himalaya, including parts of India, Nepal and Myanmar. The taxa found further east into China and Indochina regions should be referred to as *M.alticraniatus* or the allied taxa *M.thaianus*, *M.phanluongi* and *M.badius*.

**Figure 9.**
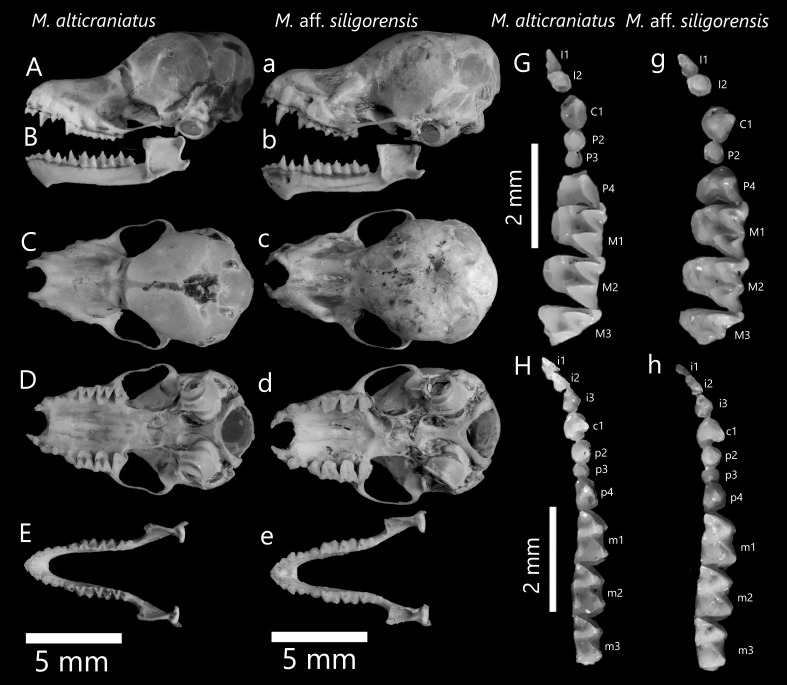
*Myotisalticraniatus* (left) and M.aff.siligorensis (right): lateral view (A, a), dorsal view (C, c) and ventral view (D, d) of cranium; lateral view (B, b) and dorsal view (E, e) of mandible; occlusal view of left upper (G, g) and right lower (H, h) toothrows.

In comparison to *M.alticraniatus*, these two smallest Vietnamese *Myotis* in our study have relatively similar cranial appearances, although these two specimens of M.aff.siligorensis exhibit significantly greater craniodental characteristics and a dissimilar dentition formula (Fig. 5, Table 2). Cranial morphologies differentiate interspecies; the braincase of M.aff.siligorensis is more robust and globular, likewise the rostrum. The zygomatic arches of M.aff.siligorensis curve evenly outwards, while those of *M.alticraniatus* curve quite deeply inwards and are more slender (Fig. 9). The lambda transition of M.aff.siligorensis is clearly observable, slightly elevated against the smooth surface of the cranium, while this feature is absent or very faint amongst specimens of *M.alticraniatus*. The dental morphologies are not clearly distinguishable between *M.alticraniatus* and M.aff.siligorensis. In *M.alticraniatus*, the upper canines C^1^ are short, with a height equivalent to that of P^4^, whereas in M.aff.siligorensis, the C^1^ are robust, measuring significantly 1.4 times higher than P^4^ (Fig. 9). In particular, P^3^ of M.aff.siligorensis is absent, resulting in the distance from the posterior of C^1^ to the anterior of P^2^ being significantly smaller than this distance observed in *M.alticraniatus* (Fig. 9, Table 2). The mandible's appearances are more elegant than in *M.alticraniatus*, though the feature of lower incisors is greater and higher than that of M.aff.siligorensis. The lower canines and premolars of *M.alticraniatus* are short and blunt, while those of M.aff.siligorensis are thinner and more pointed.

Due to the noticeable differences in craniodental morphology compared to the other 14 *Myotis* species in this study, further analysis of the taxonomy of these two Myotisaff.siligorensis specimens is needed. Simultaneously, PCA analyses are necessary to be conducted with three Vietnamese mouse-eared bats with comparable size to Myotisaff.siligorensis, which are not reported in this study due to insufficient specimens, namely: *Myotisphanluongi*, *M.ancricola* and *M.annatessae* in subsequent taxonomic investigations.

### Medium-sized Myotis

In group M, PCA results indicated dentition features, namely: CP^4^L, C^1^C^1^W, PWC^1^C^1^ and CPH, contributed the most differences amongst species, considering the interference between two first principal components (Table 3). The bivariate plots represent the exclusive species *M.altarium* as completely distinct from the other medium-sized *Myotis* species in both PC 1 and PC 2 (Fig. 8c). In addition, PC 2 of the seven medium-size *Myotis* species can distinguish them into three subgroups: A (*M.altarium*), B (*M.muricola*, *M.ater* and *M.montivagus*) and C (*M.laniger*, *M.horsfieldii* and *M.hasseltii*). These results coincided with the cranial morphological characteristics, with subgroup A characterised by a distinct skull appearance, notably featuring steep frontal and parietal lobe bone regions with significantly deep rostrum concavity and a much greater angle between the maxilla and mandible compared to other medium-sized *Myotis* bats (Fig. 5). Subgroup B consisted of flattened skulls with the lowest slope corresponding to the rostrum of the three species, while the last subgroup C was characterised by bulbous, domed and robust skulls. CPH measurements were distinctive in *M.montivagus*, separating it from the rest and, indeed, it was considered the largest of the medium-sized *Myotis* (Table 2). All the greatest difference characteristics specified *M.muricola* as being smaller than *M.ater* (One-way ANOVA, *p* < 0.01 for each of those pairs) without significant crossover (Fig. 8c). Regarding the width of canines, the coronoid process showed that *M.laniger* was significantly lower than *M.horsfieldii*, though the length of the upper canine-premolar showed the reverse tendency (Table 2). The ratio CPH/PWC^1^C^1^ was the main feature showing the difference between *M.ater* and *M.horsfieldii*, while CP^4^L and C^1^C^1^W almost intersected between them. PWC^1^C^1^ was an index that did not vary widely within medium-sized *Myotis*, but the massive appearance of *M.horsfieldii* was basically influenced by the robust and long upper canine. In this study, *Myotishasseltii* was the only *Myotis* not showing considerable differences with other species in the correlation between craniodental characters, probably as a result of insufficient specimen data. However, in general, the two *M.hasseltii* specimens were relatively large in size. The PC 1 distinguished the significantly larger *M.altarium* from the other smaller species (Fig. 8c), although the dentition width and CPH measurements did not show clarified variances and partially overlapped with the related interspecies subgroup C (*M.hasseltii* and *M.montivagus*) (Fig. 5, Table 2).

### Large-sized Myotis

In group L, dentition characteristics (CP^4^L, PWC^1^C^1^, CPH and cm_3_L) contributed the most differences, representing *Myotispilosus* as being much greater than the others. Likewise, *M.pilosus* is distinguished by a distinctly larger cranium size and skull appearance compared to the other two species in the group (Fig. 5). Although the study is limited by having only one specimen of *M.annectans*, the distinctions in descriptive statistical analysis (Table 2) and PCA (Fig. 8d) cannot negate the noticeable differences between the two large-sized *Myotis* (*M.annectans* and *M.indochinensis*) in several morphological characteristics. *Myotisannectans* was characterised by having a significantly smaller CPH value compared to *M.indochinensis* and *M.pilosus*, further making it the only individual in the large-sized *Myotis* group that is completely separate and non-intersecting in the correlation between CPH and PC 1 score (Table 2). However, in the contrary direction, measurements of CP^4^L, PWC^1^C^1^ and cm_3_L indicated *M.annectans* did not differ from *M.indochinensis*. The similarities between these two species were mentioned in the study by Nguyen et al. (2013). Based on the morphological analysis in this study, we further compared the craniodental morphology of these two *Myotis* species as follows:

Cranial morphology: The skull and mandible sizes of both species almost completely overlap (Table 2). Due to PC parameters, *M.annectans* and *M.indochinensis* entirely overlapped (Fig. 8d, Table 4). This overlap is partly explained by the fact that only one specimen of *M.annectans* was available for analysis in this study and there was insufficient data to confirm two distinct species. The braincase of *M.annectans* is slightly larger than that of *M.indochinensis*, more spherical in shape and greater in volume (Suppl. material 2). Additionally, the rostrum of *M.annectans* is more massive and shortened than that of *M.indochinensis*. *M.annectans* is further distinguishable from *M.indochinensis* by its narrower ante-orbital bridge. Sagittal, occipital and lambdoidal crests are present in *M.indochinensis*; the sagittal crest is well-developed and emerging, the lambda is distinctly pronounced and protruding with a small triangular shape and occipital and lambdoidal crests are relatively developed and visible from both the dorsal and ventral aspects of the cranium. In contrast, these characteristics in *M.annectans* are poorly developed and not noticeably expressed. The zygomatic arch of *M.indochinensis* has a broader diameter and tends to curve inwards, while that of other species is smooth and evenly bent outwards.

Dental morphology: The dentition of *Myotisannectans* tends to shorten and widen, while in *M.indochinensis*, the teeth are more robust, taller and more pointed. The canines and large premolars are more developed in *M.indochinensis* compared to *M.annectans*. The c_1_ of *M.annectans* are insignificantly shorter in height and barely exceed the height of p_4_, while the c_1_ of *M.indochinensis* is pointier, approximately reaching the height of p_4_. The most basic characteristic that distinguishes these two species is that *M.annectans* has reduced dentition, with P^3^ and p_3_ of both the maxilla and mandible being typically absent, although they remain intact in *M.indochinensis*.

## Conclusions

Our analyses demonstrated craniodental morphology variations of 15 *Myotis* species in Vietnam, which could be divided into four group clusters with distinct sizes: small-sized, medium-sized, large-sized, extra-large-sized, all with significant interspecific variances. Multivariate analyses also specified noteworthy differentiations in craniodental morphology based on the principal measurements: P^4^M^3^L, ZYW, C^1^C^1^W, CPH, cm_3_L, cp_4_L, p_4_m_3_L, m_1_m_3_L, CP^4^L, PWC^1^C^1^ and CPH, which contributed the most to the interspecific craniodental variation in Vietnamese mouse-eared bats. Simultaneously, we revealed two specimen of Myotisaff.siligorensis with distinct craniodental morphology that could be listed in the "*Myotissiligorensis*" complex. Furthermore, our study established comparisons between morphometrically similar species according to patterns of morphological differences in the study area, which will be helpful for classifying and constructing a comprehensive craniodental morphometric identification key for all species of the genus *Myotis* in Vietnam and neighbouring southeast Asian regions in further research.

## Supplementary Material

3AFDE375-79BF-5E7C-BD29-1B0F343E5BC3 10.3897/BDJ.12.e122597.suppl1 Supplementary material 1 List of species and collection data Data type Table Brief description List of species, number of specimens and collected sample localities recorded in this study. File: oo_1049025.xlsx https://binary.pensoft.net/file/1049025 Huong Yen Vu, Truong Son Nguyen, Tuan Hai Bui, Trung Thanh Hoang, Kim Luong Vu

D5022279-F906-5AD5-9DDB-07F18B4D2528 10.3897/BDJ.12.e122597.suppl2 Supplementary material 2 *Myotisannectans* and *Myotisindochinensis*, cranium, mandible and toothrows. Data type Image Brief description Lateral view (A, a), dorsal view (C, c) and ventral view (D, d) of cranium; lateral view (B, b) and dorsal views (E, e) of mandible; occlusal view of left upper (G, g) and right lower (H, h) toothrows. File: oo_1044096.png https://binary.pensoft.net/file/1044096 Huong Yen Vu, Truong Son Nguyen, Tuan Hai Bui, Trung Thanh Hoang, Kim Luong Vu

## Figures and Tables

**Table 1.** List of craniodental measurements used in this study.

**Character**	**Explanation**
**Cranium**	
STOTL	Total length of the skull (from the anterior rim of the alveolus of the first upper incisor to the most projecting point of the occipital region).
GTL	Greatest length of skull (from the front of the 1st upper incisor to the most projecting point of the occipital region).
CCL	Condylo-canine length (distance from the exoccipital condyle to the most anterior part of the canine).
CM^3^L	Maxillary toothrow length (distance from the front of upper canine to the back of the crown of the third molar).
CP^4^L	Upper canine-premolar length (from the front of the upper canine to the back of the crown of the last premolar).
P^4^M^3^L	Upper molariform toothrow length (from the posterior upper premolar to the last molar).
M^1^M^3^L	Upper molar crown length (from the front of the 1st upper molar to the last molar).
MAW	Mastoid width (greatest distance across the mastoid region).
BCH	Braincase height (from the basisphenoid at the level of the hamular processes to the highest part of the skull, including the sagittal crest, if present).
BB	Breadth of braincase at the posterior roots of zygomatic arches.
GBCW	Greatest width of the braincase.
IOW	Interorbital width (least width of the interorbital constriction).
ZYW	Zygomatic width (greatest width of the skull across the zygomatic arches).
PWC^1^C^1^	Anterior palatal width (least distance between the inner borders of the upper canines).
PWM^3^M^3^	Posterior palatal width (least distance between the inner borders of the last upper molars).
C^1^C^1^W	Width across the upper canines (greatest width across the outer borders of the upper canines).
M^3^M^3^W	Width across the upper molars (greatest width across the outer crowns of the last upper molars).
**Mandible**	
ML	Mandible length (distance from the anterior rim of the alveolus of the first lower incisor to the most posterior part of the condyle).
CPH	Least height of the coronoid process (distance from the tip of the coronoid process to the apex of the indentation on the inferior surface of the ramus adjacent to the angular process).
cm_3_L	Mandibular tooth row length (distance from the front of the lower canine to the back of the crown of the third lower molar).
cp_4_L	Lower canine-premolar length (distance from the front of the lower canine to the back of the crown of the posterior premolar).
p_4_m_3_L	Lower molariform toothrow length (Posterior lower premolar to the last lower molar length).
m_1_m_3_L	Lower molars crown length.

**Table 2.** Minimum, maximum in the upper row and mean and standard deviation (if n ≥ 2) in the bottom row of 23 craniodental measurements in 15 mouse-eared bat species from Vietnam.

**Character**	***M.alticraniatus***	***M.rosseti***	**M.aff.siligorensis**	***M.annamiticus***	***M.montivagus***
**N**	**19**	**2**	**2**	**5**	**1**
STOTL	11.86 - 12.29	12.33 - 12.65	12.4 - 12.81	13.01 - 13.77	14.97
	12.06 ± 0.12	12.49 ± 0.23	12.61 ± 0.29	13.4 ± 0.33	
GTL	11.92 - 12.53	12.54 - 12.86	12.64 - 12.91	13.29 - 13.91	15.33
	12.21 ± 0.16	12.7 ± 0.23	12.78 ± 0.19	13.65 ± 0.3	
CCL	9.09 - 9.66	9.48 - 9.58	9.71 - 9.93	10.06 - 10.67	12.03
	9.38 ± 0.15	9.53 ± 0.07	9.82 ± 0.16	10.37 ± 0.29	
CM^3^L	4.33 - 4.67	4.34 - 4.47	4.47 - 4.62	4.97 - 5.16	5.76
	4.46 ± 0.1	4.41 ± 0.09	4.55 ± 0.11	5.09 ± 0.07	
CP^4^L	1.75 - 2.21	1.69 - 1.76	1.84 - 1.89	2.41 - 2.61	2.77
	2.04 ± 0.12	1.73 ± 0.05	1.87 ± 0.04	2.53 ± 0.08	
P^4^M^3^L	3.08 - 3.31	3.27 - 3.44	3.38 - 3.51	3.34 - 3.51	4.44
	3.18 ± 0.07	3.36 ± 0.12	3.45 ± 0.09	3.42 ± 0.07	
M^1^M^3^L	2.54 - 2.78	2.77 - 2.81	2.75 - 2.92	2.67 - 2.83	3.56
	2.66 ± 0.06	2.79 ± 0.03	2.84 ± 0.12	2.78 ± 0.06	
MAW	6.15 - 6.72	6.87 - 6.93	6.58 - 6.69	6.48 - 6.98	7.9
	6.39 ± 0.15	6.9 ± 0.04	6.64 ± 0.08	6.77 ± 0.2	
BCH	4.39 - 5.07	4.8	4.93 - 4.98	4.93 - 5.34	5.46
	4.63 ± 0.19	4.8	4.96 ± 0.04	5.15 ± 0.16	
BB	5.73 - 6.38	6.73 - 6.75	6.22 - 6.37	6.29 - 6.58	7.57
	6.02 ± 0.17	6.74 ± 0.01	6.3 ± 0.11	6.44 ± 0.12	
GBCW	5.71 - 6.37	6.35 - 6.48	6.47 - 6.54	6.54 - 6.81	7.13
	5.96 ± 0.15	6.42 ± 0.09	6.51 ± 0.05	6.67 ± 0.11	
IOW	2.13 - 3.15	3.35	3.12 - 3.22	3.16 - 3.32	3.65
	2.94 ± 0.22	3.35	3.17 ± 0.07	3.25 ± 0.08	
ZYW	6.87 - 7.84	8.05 - 8.36	7.42 - 7.49	7.19 - 7.67	10.09
	7.1 ± 0.23	8.21 ± 0.22	7.46 ± 0.05	7.47 ± 0.19	
PWC^1^C^1^	1.93 - 2.38	2.11 - 2.15	2.41 - 2.54	2.33 - 2.59	2.33
	2.1 ± 0.11	2.13 ± 0.03	2.48 ± 0.09	2.49 ± 0.11	
PWM^3^M^3^	2.31 - 2.8	2.71 - 2.79	2.61 - 2.76	2.69 - 2.95	3.31
	2.56 ± 0.13	2.75 ± 0.06	2.69 ± 0.11	2.84 ± 0.11	
C^1^C^1^W	2.66 - 3.06	3.24 - 3.44	3.45 - 3.54	3.1 - 3.45	3.91
	2.95 ± 0.1	3.34 ± 0.14	3.5 ± 0.06	3.3 ± 0.15	
M^3^M^3^W	4.55 - 5.02	5.06 - 5.24	4.98 - 5.05	4.92 - 5.11	6.46
	4.77 ± 0.14	5.15 ± 0.13	5.02 ± 0.05	5.03 ± 0.07	
ML	8.24 - 8.68	8.63 - 9.11	8.78 - 9.07	9.14 - 9.58	11.63
	8.52 ± 0.11	8.87 ± 0.34	8.93 ± 0.21	9.39 ± 0.18	
CPH	2.05 - 2.26	2.6 - 2.61	2.29 - 2.35	2.16 - 2.58	3.74
	2.14 ± 0.07	2.61 ± 0.01	2.32 ± 0.04	2.35 ± 0.15	
cm_3_L	4.38 - 4.82	4.52 - 4.65	4.61 - 5.03	5.05 - 5.36	6.45
	4.62 ± 0.11	4.59 ± 0.09	4.82 ± 0.3	5.26 ± 0.13	
cp_4_L	1.69 - 2.01	1.61 - 1.62	1.83 - 1.93	1.98 - 2.46	2.38
	1.79 ± 0.09	1.62 ± 0.01	1.88 ± 0.07	2.28 ± 0.18	
p_4_m_3_L	3.11 - 3.62	3.42 - 3.56	3.61 - 3.69	3.53 - 3.65	4.7
	3.38 ± 0.1	3.49 ± 0.1	3.65 ± 0.06	3.58 ± 0.05	
m_1_m_3_L	2.68 - 2.95	2.87 - 3.06	3.09 - 3.15	2.87 - 3.06	3.76
	2.84 ± 0.08	2.97 ± 0.13	3.12 ± 0.04	2.98 ± 0.08	
**Character**	***M.muricola***	***M.laniger***	***M.ater***	***M.horsfieldii***	***M.altarium***
**N**	**30**	**85**	**26**	**13**	**1**
STOTL	13.09 - 14.04	13.36 - 14.54	14.04 - 15.09	13.85 - 15.1	15.19
	13.52 ± 0.22	14.09 ± 0.26	14.48 ± 0.27	14.61 ± 0.37	
GTL	13.39 - 14.32	13.81 - 14.78	14.31 - 15.34	13.98 - 15.36	15.69
	13.81 ± 0.2	14.33 ± 0.22	14.78 ± 0.25	14.88 ± 0.41	
CCL	10.48 - 11.21	10.65 - 11.69	11.21 - 12.11	11.01 - 12.05	12.45
	10.8 ± 0.19	11.24 ± 0.21	11.6 ± 0.21	11.51 ± 0.28	
CM^3^L	4.97 - 5.33	5.25 - 5.8	5.23 - 5.81	5.28 - 5.68	6.21
	5.18 ± 0.1	5.56 ± 0.11	5.58 ± 0.13	5.53 ± 0.14	
CP^4^L	2.16 - 2.62	2.51 - 2.95	2.21 - 2.7	2.27 - 2.79	3.15
	2.4 ± 0.1	2.69 ± 0.09	2.49 ± 0.11	2.55 ± 0.13	
P^4^M^3^L	3.53 - 3.99	3.66 - 4.11	3.96 - 4.46	3.81 - 4.12	4.24
	3.81 ± 0.11	3.87 ± 0.1	4.19 ± 0.12	3.95 ± 0.1	
M^1^M^3^L	2.89 - 3.34	2.91 - 3.31	3.25 - 3.62	3.13 - 3.39	3.44
	3.14 ± 0.09	3.15 ± 0.08	3.45 ± 0.09	3.24 ± 0.08	
MAW	6.49 - 7.38	6.78 - 7.47	7.09 - 7.97	7.35 - 7.91	8.23
	7.09 ± 0.18	7.16 ± 0.15	7.54 ± 0.21	7.66 ± 0.19	
BCH	4.42 - 5.15	4.88 - 5.92	4.95 - 5.59	5.14 - 5.87	5.95
	4.85 ± 0.16	5.38 ± 0.18	5.31 ± 0.16	5.54 ± 0.24	
BB	6.55 - 7.14	6.43 - 7.28	6.86 - 7.62	7.15 - 7.65	8.26
	6.87 ± 0.16	6.74 ± 0.17	7.37 ± 0.2	7.36 ± 0.14	
GBCW	6.01 - 6.74	6.73 - 7.37	6.37 - 7.05	7.05 - 7.58	7.85
	6.35 ± 0.17	7.06 ± 0.12	6.78 ± 0.19	7.31 ± 0.18	
IOW	3.18 - 3.54	3.18 - 3.76	3.23 - 3.94	3.37 - 3.81	4.74
	3.37 ± 0.08	3.39 ± 0.11	3.57 ± 0.17	3.62 ± 0.12	
ZYW	7.88 - 9.05	7.75 - 8.59	8.99 - 9.74	8.68 - 9.38	10.01
	8.68 ± 0.25	8.19 ± 0.17	9.46 ± 0.2	8.98 ± 0.22	
PWC^1^C^1^	1.74 - 2.38	2.14 - 2.68	1.95 - 2.61	2.16 - 2.68	2.67
	2 ± 0.14	2.34 ± 0.11	2.25 ± 0.14	2.47 ± 0.17	
PWM^3^M^3^	2.71 - 3.28	2.73 - 3.32	2.88 - 3.26	2.97 - 3.34	3.63
	2.97 ± 0.13	2.99 ± 0.11	3.1 ± 0.09	3.18 ± 0.11	
C^1^C^1^W	3.21 - 3.71	3.22 - 3.79	3.62 - 4.23	3.71 - 4.34	3.92
	3.47 ± 0.13	3.53 ± 0.12	4.02 ± 0.12	4.06 ± 0.2	
M^3^M^3^W	5.22 - 5.9	5.13 - 5.76	5.87 - 6.28	5.55 - 6.08	6.62
	5.62 ± 0.17	5.46 ± 0.15	6.06 ± 0.12	5.81 ± 0.16	
ML	9.65 - 10.36	9.76 - 11.01	10.43 - 11.44	9.99 - 11.11	11.85
	10.04 ± 0.22	10.28 ± 0.22	10.92 ± 0.22	10.61 ± 0.34	
CPH	2.65 - 3.17	2.55 - 2.97	3.13 - 3.62	2.82 - 3.28	3.61
	2.95 ± 0.13	2.72 ± 0.09	3.37 ± 0.13	3.08 ± 0.15	
cm_3_L	4.11 - 5.91	5.14 - 6.29	5.69 - 6.43	5.51 - 6.12	6.68
	5.45 ± 0.29	5.73 ± 0.17	5.92 ± 0.17	5.87 ± 0.17	
cp_4_L	1.91 - 2.48	2 - 2.54	2.09 - 2.45	2.15 - 2.62	3.06
	2.15 ± 0.12	2.33 ± 0.11	2.29 ± 0.1	2.4 ± 0.12	
p_4_m_3_L	3.81 - 4.36	3.87 - 4.3	4.13 - 4.6	3.96 - 4.54	4.59
	4.03 ± 0.13	4.11 ± 0.09	4.41 ± 0.11	4.24 ± 0.16	
m_1_m_3_L	3.22 - 3.52	3.13 - 3.73	3.44 - 3.91	3.35 - 3.73	3.87
	3.37 ± 0.07	3.41 ± 0.09	3.69 ± 0.11	3.5 ± 0.11	
**Character**	***M.hasseltii***	***M.annectans***	***M.indochinensis***	***M.pilosus***	***M.chinensis***
**N**	**2**	**1**	**15**	**3**	**4**
STOTL	15.62 - 15.67	17.69	16.96 - 17.98	19.41 - 20.24	22.19 - 24.61
	15.65 ± 0.04		17.51 ± 0.36	19.83 ± 0.42	23.54 ± 1
GTL	15.87 - 16.01	17.91	17.41 - 18.52	19.68 - 20.51	22.81 - 25.01
	15.94 ± 0.1		18.04 ± 0.37	20.11 ± 0.42	23.97 ± 0.9
CCL	12.11 - 12.29	14.33	13.8 - 14.72	15.9 - 16.29	18.83 - 19.68
	12.2 ± 0.13		14.33 ± 0.28	16.1 ± 0.2	19.36 ± 0.37
CM^3^L	5.72 - 5.87	6.99	6.86 - 7.41	8.01 - 8.37	9.57 - 9.97
	5.8 ± 0.11		7.13 ± 0.17	8.18 ± 0.18	9.78 ± 0.2
CP^4^L	2.49 - 2.82	3.34	2.89 - 3.41	3.57 - 3.84	4.53 - 4.79
	2.66 ± 0.23		3.17 ± 0.15	3.67 ± 0.15	4.69 ± 0.11
P^4^M^3^L	3.93 - 4.33	5.21	5.09 - 5.42	5.81 - 6.14	6.37 - 7.05
	4.13 ± 0.28		5.27 ± 0.11	5.95 ± 0.17	6.7 ± 0.31
M^1^M^3^L	3.43 - 3.54	4.22	4.18 - 4.53	4.81 - 5.02	4.96 - 5.69
	3.49 ± 0.08		4.3 ± 0.09	4.89 ± 0.11	5.45 ± 0.33
MAW	8.21 - 8.27	8.98	8.64 - 9.63	9.66 - 10.11	12.01 - 12.14
	8.24 ± 0.04		9.05 ± 0.3	9.91 ± 0.23	12.08 ± 0.05
BCH	6.05 - 6.24	5.86	5.81 - 6.68	6.64 - 7.04	8.31 - 9.21
	6.15 ± 0.13		6.27 ± 0.26	6.81 ± 0.21	8.77 ± 0.42
BB	7.76 - 7.98	8.87	8.56 - 9.42	9.16 - 9.62	11.06 - 11.59
	7.87 ± 0.16		9.07 ± 0.25	9.43 ± 0.24	11.37 ± 0.22
GBCW	7.48 - 8.03	8.29	7.53 - 8.35	9.26 - 9.84	10.44 - 10.57
	7.76 ± 0.39		7.89 ± 0.24	9.58 ± 0.29	10.51 ± 0.05
IOW	3.8 - 4.08	4.34	4.17 - 4.66	4.85 - 4.86	5.29 - 5.57
	3.94 ± 0.2		4.38 ± 0.14	4.86 ± 0.01	5.45 ± 0.12
ZYW	9.61 - 9.82	11.48	11.57 - 12.71	12.34 - 12.77	15.81 - 16.22
	9.72 ± 0.15		11.91 ± 0.3	12.62 ± 0.25	15.98 ± 0.19
PWC^1^C^1^	2.33 - 2.51	2.86	2.37 - 3.33	3.29 - 3.85	3.73 - 3.91
	2.42 ± 0.13		2.77 ± 0.22	3.58 ± 0.28	3.83 ± 0.08
PWM^3^M^3^	3.2 - 3.51	3.91	3.64 - 4.28	3.92 - 4.43	4.62 - 5.03
	3.36 ± 0.22		4.01 ± 0.14	4.18 ± 0.26	4.85 ± 0.21
C^1^C^1^W	4.19 - 4.21	4.98	4.75 - 5.18	5.33 - 5.69	5.89 - 6.48
	4.2 ± 0.01		4.94 ± 0.16	5.54 ± 0.19	6.18 ± 0.27
M^3^M^3^W	6.07 - 6.38	7.56	7.63 - 8.06	7.57 - 8.23	9.3 - 10.06
	6.23 ± 0.22		7.76 ± 0.12	7.98 ± 0.36	9.69 ± 0.35
ML	10.95 - 11.39	13.28	12.96 - 14.03	14.9 - 15.51	18.52 - 18.91
	11.17 ± 0.31		13.64 ± 0.32	15.18 ± 0.31	18.68 ± 0.17
CPH	3.34 - 3.46	4.08	4.11 - 4.67	4.41 - 4.72	6.16 - 6.39
	3.4 ± 0.08		4.44 ± 0.16	4.56 ± 0.16	6.25 ± 0.1
cm_3_L	6.15 - 6.35	7.45	7.27 - 7.89	8.73 - 8.85	10.17 - 10.68
	6.25 ± 0.14		7.59 ± 0.17	8.8 ± 0.06	10.41 ± 0.27
cp_4_L	2.45 - 2.6	2.95	2.81 - 3.22	3.34 - 3.72	4.21 - 4.31
	2.53 ± 0.11		2.99 ± 0.11	3.48 ± 0.21	4.25 ± 0.05
p_4_m_3_L	4.4 - 4.58	5.72	5.39 - 5.97	6.19 - 6.31	7.16 - 7.81
	4.49 ± 0.13		5.63 ± 0.14	6.26 ± 0.06	7.44 ± 0.29
m_1_m_3_L	3.71 - 3.82	4.66	4.47 - 4.83	5.17 - 5.35	5.86 - 6.43
	3.77 ± 0.08		4.67 ± 0.1	5.25 ± 0.09	6.05 ± 0.26

**Table 3.** Character loadings for log-transformed measurements (PCs 1, 2) of 15 Vietnamese *Myotis*, Group S, Group M and Group L.

**Character**	**All Taxa**		**Group S**		**Group M**		**Group L**	
	**PC 1**	**PC 2**	**PC 1**	**PC 2**	**PC 1**	**PC 2**	**PC 1**	**PC 2**
STOTL	0.179	0.072	0.183	0.012	0.146	0.087	0.212	0.077
GTL	0.183	0.054	0.193	0.019	0.152	0.072	0.189	0.089
CCL	0.197	0.063	0.175	-0.019	0.149	0.081	0.197	0.068
CM^3^L	0.214	0.161	0.215	-0.120	0.119	0.181	0.231	0.136
CP^4^L	0.209	**0.421**	**0.354**	-**0.588**	0.036	**0.383**	0.225	**0.409**
P^4^M^3^L	**0.221**	-0.069	0.134	0.084	0.193	-0.029	0.203	0.145
M^1^M^3^L	0.215	-0.118	0.081	0.111	0.205	-0.079	0.210	0.179
MAW	0.173	-0.030	0.114	0.142	0.194	0.003	0.163	-0.132
BCH	0.157	**0.244**	0.206	0.078	0.162	0.305	0.160	0.016
BB	0.186	-0.148	0.127	0.199	0.237	-0.096	0.075	-0.068
GBCW	0.145	**0.267**	0.201	0.140	0.118	0.319	0.319	-0.070
IOW	0.181	-0.033	0.227	**0.297**	0.208	0.017	0.173	0.012
ZYW	**0.235**	-0.283	0.110	**0.287**	**0.287**	-0.247	0.112	-0.052
PWC^1^C^1^	0.161	**0.430**	**0.320**	0.085	0.199	**0.511**	**0.448**	-**0.727**
PWM^3^M^3^	0.191	-0.038	0.204	0.156	0.168	0.019	0.089	-0.194
C^1^C^1^W	**0.228**	-0.141	0.213	0.221	**0.354**	-0.050	0.196	-0.010
M^3^M^3^W	0.213	-0.193	0.102	0.139	0.234	-0.142	0.059	-0.003
ML	0.217	-0.005	0.170	0.022	0.191	0.017	0.188	0.052
CPH	**0.313**	-**0.432**	0.195	**0.339**	**0.405**	-**0.390**	0.089	0.195
cm_3_L	**0.228**	0.096	0.228	-0.103	0.179	0.118	**0.248**	0.061
cp_4_L	**0.233**	**0.287**	**0.426**	-**0.360**	0.156	0.277	**0.261**	0.191
p_4_m_3_L	**0.229**	-0.053	0.123	0.050	0.193	-0.015	0.175	0.053
m_1_m_3_L	**0.222**	-0.081	0.105	0.073	0.193	-0.042	0.187	0.122
% ***Variance***	***91.36***	***3.68***	***59.63***	***19.49***	***48.73***	***25.76***	***75.24***	***8.55***

**Table 4.** Descriptive statistics of first two principal components of 15 studied *Myotis* in Vietnam.

**Species**	**PC1**	**PC2**
*M.alticraniatus*	–1.73 - –1.34	–0.72 - 0.38
	–1.52 ± 0.11	–0.09 ± 0.28
*M.rosseti*	–1.25 - –1.06	–2.11 - –2.1
	–1.16 ± 0.13	–2.11 ± 0.003
M.aff.siligorensis	–1.13 - –1.02	–0.33 - –0.09
	–1.07 ± 0.08	–0.21 ± 0.17
*M.muricola*	–0.71 - –0.23	–1.87 - –0.51
	–0.38 ± 0.11	–1.24 ± 0.34
*M.laniger*	–0.45 - 0.01	0.38 - 1.31
	–0.19 ± 0.11	0.86 ± 0.22
*M.ater*	–0.06 - 0.43	–1.84 - –0.68
	0.23 ± 0.12	–1.27 ± 0.303
*M.horsfieldii*	0.23 ± 0.12	–0.49 - 0.52
	0.16 ± 0.15	0.03 ± 0.33
*M.montivagus*	0.59	–1.1296
*M.hasseltii*	0.59 - 0.66	0.23 - 0.06
	0.63 ± 0.046	0.08 ± 0.21
*M.altarium*	0.97	0.76
*M.annectans*	1.8	–0.16
*M.indochinensis*	1.68 - 2.09	–1.34 - –0.21
	1.89 ± 0.12	–0.82 ± 0.28
*M.pilosus*	2.53 - 2.81	0.97 - 1.13
	2.68 ± 0.14	1.07 ± 0.08
*M.chinensis*	3.78 - 4.12	1.11 - 1.27
	3.99 ± 0.15	1.19 ± 0.07

**Table 5.** Significance level when comparing PC1 and PC2 scores between species of *Myotis*.

	*M.hasseltii*	*M.altarium*	*M.montivagus*	*M.horsfieldii*	*M.ater*	*M.laniger*	*M.muricola*
*M.hasseltii*		s, s	n, s	s, n	s, s	s, n	s, s
*M.altarium*			s, s	s, s	s, s	s, s	s, s
*M.montivagus*				s, s	s, n	s, s	s, n
*M.horsfieldii*					n, s	s, n	s, s
*M.ater*						s, s	s, n
*M.laniger*							n, s
*M.muricola*							
